# Digital Path Approach Despeckle Filter for Ultrasound Imaging and Video

**DOI:** 10.1155/2017/9271251

**Published:** 2017-10-08

**Authors:** Marek Szczepański, Krystian Radlak

**Affiliations:** Faculty of Automatic Control, Electronics and Computer Science, Silesian University of Technology, Akademicka 16, 44-100 Gliwice, Poland

## Abstract

We propose a novel filtering technique capable of reducing the multiplicative noise in ultrasound images that is an extension of the denoising algorithms based on the concept of digital paths. In this approach, the filter weights are calculated taking into account the similarity between pixel intensities that belongs to the local neighborhood of the processed pixel, which is called a path. The output of the filter is estimated as the weighted average of pixels connected by the paths. The way of creating paths is pivotal and determines the effectiveness and computational complexity of the proposed filtering design. Such procedure can be effective for different types of noise but fail in the presence of multiplicative noise. To increase the filtering efficiency for this type of disturbances, we introduce some improvements of the basic concept and new classes of similarity functions and finally extend our techniques to a spatiotemporal domain. The experimental results prove that the proposed algorithm provides the comparable results with the state-of-the-art techniques for multiplicative noise removal in ultrasound images and it can be applied for real-time image enhancement of video streams.

## 1. Introduction

Medical ultrasound is an imaging technique widely used in the diagnosis and assessment of internal body structures, and it plays a key role in treating various diseases. Compared to the other techniques of medical imaging, it is safe, noninvasive, and well tolerated by the patient, and ultrasound images are captured in real time at reasonable price. An accurate analysis of ultrasound images and thus an appropriate diagnosis are difficult due to the fact that the images are contaminated with characteristic granular structures called speckle noise, which deteriorates contrast and hinders the identification of important image details [1]. Although the ultrasound images are subjected to an initial improvement during the acquisition process, their quality is still far from optimal. Therefore, the main aim of image denoising is to remove the noise, while preserving the important details.

Recently, a plethora of methods capable of diminishing speckle noise have been proposed [2]. According to the works presented in [3–5], one of the most promising results for ultrasound images was obtained with algorithms based on anisotropic diffusion techniques [6] and the idea of nonlocal means [7–11]. However, majority of them were designed for static images, and not much attempt has been made to video by considering temporal coherence.

Video sequence processing algorithms can take an advantage of high correlation between adjacent frames, exploring spatial and temporal neighborhood. These properties are incorporated using different variants of averaging the pixel intensities in successive video frames. Simple temporal filters, such as temporal Gaussian, efficiently remove noise for videos with slowly moving objects, but averaging operation of subsequent frames can cause “ghosting” artifacts in the output results. Some works indicate that motion compensation allows us to reduce the blurring effect [12, 13]. Additionally, “ghosting” effects can be reduced by application of the statistical models that reflects the joint distributions of wavelet coefficients over space and time [14, 15]. Ghosting artifacts may be also omitted using temporal version of a bilateral filter that was successfully applied in the adaptive spatiotemporal accumulation (ASTA) filter [16].

In our previous works on video filtering, a spatiotemporal denoising scheme utilized local switching between spatial digital path approaches and temporal Gaussian filtering [17] and a fuzzy spatiotemporal filter that was later described [18]. Another interesting study connected with video denoising introduces a 3D filtering framework that is based on fuzzy set logic to combine the gradient values in different directions between previous and current temporal frames [19].

The main aim of this research is to develop a filter that will efficiently cope with multiplicative noise in ultrasound images and videos. The proposed algorithm is based on the idea of spatial digital paths presented in [20, 21]. The original 2D algorithm presented in [20] perfectly removes Gaussian noise and after some modifications impulsive noise but fails in the presence of multiplicative interferences. The new denoising scheme explores spatial or spatiotemporal pixel neighborhood in order to calculate the filter weights for pixels belonging to the processing window. Digital paths in a spatiotemporal domain could be understood as trajectories or object displacements in subsequent frames of a video stream.

The proposed technique utilizes a specific kind of digital paths, the so called *escaping paths*, and extends this concept from the spatial domain (2D) to the spatiotemporal domain (3D) that allows us to efficiently reduce the speckle noise. Furthermore, to increase the filter denoising ability, the new method of path creation utilizes the extended neighborhood introduced by von Neumann for cellular automata [22]. A detailed description of the proposed algorithm and its extensions are presented in Section 2. In Section 3, we present the results of experiments and the comparison with competitive filters. Finally, the conclusions are drawn in Section 4.

## 2. Method

### 2.1. Speckle Noise Model

Speckle noise is a signal-dependent and non-Gaussian multiplicative image distortion. Such noise is generally more difficult to remove than additive noise [23]. This type of distortion appears in sonar, laser, and synthetic aperture radar (SAR), and it depends on the structure of the material being imaged and various acquisition parameters [24]. Speckle noise is a random process and can be modeled using gamma, Rayleigh, and Fisher-Tippett distribution [25–27]. In our work, the simplified speckle noise model has been employed, since it has been applied successfully in many studies [11, 24, 28, 29].

(1) $$\begin{array}{c} u\left(x\right)=v\left(x\right)+\sqrt{v\left(x\right)}\cdot n\left(x\right), \end{array}$$

where *u*(**x**), **v**(**x**), and **n**(**x**) denote the observed signal, original unknown signal, and zero-mean Gaussian noise, respectively.

### 2.2. General Filter Framework

Since smoothing is commonly used to decrease the level of random noise, an averaging operation is required in order to replace the noisy pixel, *v*(**x**), with a suitable pixel representative for the local spatiotemporal neighborhood of point **x** = (*x*, *y*, *t*). So the general form of the fuzzy adaptive filters in this work is defined as the weighted average of inputs inside the spatiotemporal window *W* that are in neighborhood relation *𝒩* with a center pixel **x** [30, 31].

(2) $$\begin{array}{c} \hat{v}\left(x\right)=\sum_{x_{i}\in N\left(x\right)}w_{i}u\left(x_{i}\right)=\frac{\sum_{x_{i}\in N\left(x\right)}\mu_{i}\left(x,x_{i}\right)u\left(x_{i}\right)}{\sum_{x_{i}\in N\left(x\right)}\mu_{i}\left(x,x_{i}\right)}, \end{array}$$

where *u*(**x**_*i*_) and $\hat{v}\left(x\right)$ denote filter inputs and outputs, respectively, and *μ*(**x**, **x**_*i*_) is a similarity function computed along digital path starting at window central point **x**, associated with its neighbor **x**_*i*_, and bounded by the spatiotemporal processing window *W*.

To describe the model of digital paths, a few notions should be introduced: digital lattice ℋ = (*X*, *𝒩*) defined by *X*, which is the set of all points of the image sequence, and a neighborhood relation *𝒩* between the lattice points [32].

A digital path *P* = {*p*_*i*_}_*i*=0_^*n*^ defined on the image lattice is a sequence of adjacent points (*p*_*i*_, *p*_*i*+1_) ∈ *𝒩*, and *n* is a number of path segments. Let *L*(*P*) denote the length of the digital path *P*{*p*_*i*_}_*i*=0_^*n*^ that is calculated as ∑_*i*=1_^*n*−1^*ρ*(*p*_*i*_, *p*_*i*+1_), where *ρ* denotes the spatiotemporal Euclidean distance between two adjacent points of the path.

The connection cost over the single digital path *P* = {*p*_*i*_}_*i*=0_^*n*^ can be determined as a measure of dissimilarity between image pixels *p*_0_, *p*_1_,…, *p*_*n*_ that forms a specific path linking *p*_0_ and *p*_*n*_ [33, 34]. In the new approach, the connection cost will be calculated as a combination of the topological length of the path and the cumulative differences of gray levels. Thus, the connection cost for the entire path *Λ*(*P*) can be defined as follows:

(3) $$\begin{array}{c} \Lambda \left(P\right)=\overset{n-1}{\underset{i=0}{\sum }}\left|u\left(p_{i+1}\right)-u\left(p_{i}\right)\right|\cdot \overset{n-1}{\underset{i=0}{\sum }}\rho \left(p_{i},p_{i+1}\right). \end{array}$$

### 2.3. Similarity Functions

Weights for the filter described by (2) can be defined in many ways; in our case, we use two different approaches based on connection costs calculated along digital paths. We created two kinds of membership functions which leads to the filters with slightly different properties.

#### 2.3.1. DPA_1st_

**Figure 1:**
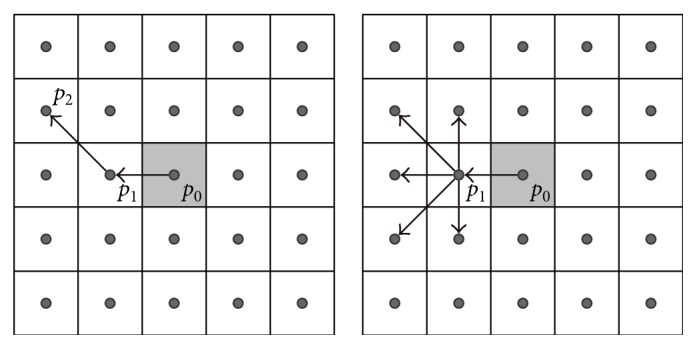
Illustration of paths created on the 2D image lattice with the DPA_1st_ approach, used to determine the similarity function between two adjacent points.

In this approach, similarity functions are defined for all neighbors of the central point **x** that remain in the neighborhood relation. To define the similarity function *μ*(**x**, **x**_*i*_) between the filtered point **x** and its *i*th neighbor, we create digital paths starting at center point **x** = *p*_0_, intersecting **x**_*i*_ = *p*_1_ and finally terminating at point *p*_*n*_, which may be reached in *n* steps from *p*_0_. The similarity function defined for points *p*_0_ and *p*_1_ uses paths exploring the further neighborhood of the central point passing points *p*_2_,…, *p*_*n*_, so that the filtering result will be better suited to local image structures. An illustration of this idea is presented in Figure 1. This approach will be further denoted as DPA_1st_. In this case, the similarity function takes the form as follows:

(4) $$\begin{array}{c} \mu \left(x,x_{i}\right)=\mu \left(p_{0},p_{1}\right)=\overset{\omega }{\underset{m=1}{\sum }}g\left(\Lambda \left\{p_{0},p_{1},p_{2}^{m},\ldots ,p_{n}^{m}\right\}\right), \end{array}$$

where *ω* denotes the number of all possible paths *P*_*m*_ = {*p*_0_, *p*_1_, *p*_2_^*m*^,…, *p*_*n*_^*m*^} with *n* steps totally included in the processing window *W*, originating at **x** = *p*_0_ and crossing **x**_*i*_ = *p*_1_; *m* is the index of a specific path; *Λ*{·} is a dissimilarity value along a specific path; and *g*(·) is a smooth function of *Λ*. In this work, the exponential function is used as *g*(·) [20], so the similarity function takes the form as follows:

(5) $$\begin{array}{c} \mu \left(x,x_{i}\right)=\mu \left(p_{0},p_{1}\right)=\overset{\omega }{\underset{m=1}{\sum }}\text{exp}\left[-\beta \cdot \Lambda \left\{p_{0},p_{1},p_{2}^{m},\ldots ,p_{n}^{m}\right\}\right], \end{array}$$

where *β* is the filter design parameter.

#### 2.3.2. DPA_last_

**Figure 2:**
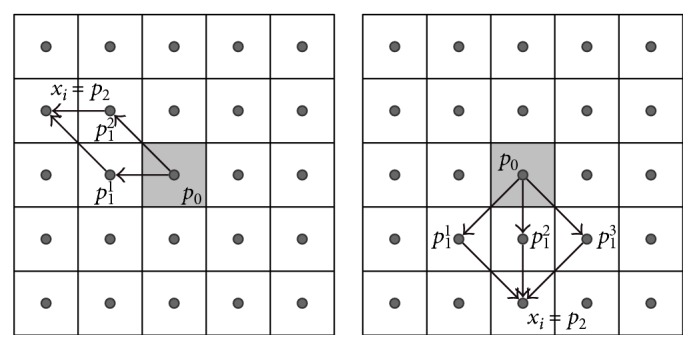
Illustration of paths created on the 2D image lattice with the DPA_last_ approach.

Another approach is to determine the similarity function between pixels **x** and **x**_*i*_ by all possible paths connecting them (Figure 2). This approach will be denoted as DPA_last_, and the similarity function between image points **x** and **x**_*i*_ can be defined as follows:

(6) $$\begin{array}{c} \mu \left(x,x_{i}\right)=\mu \left(p_{0},p_{n}\right)=\overset{\omega }{\underset{m=1}{\sum }}g\left(\Lambda \left\{p_{0},p_{1}^{m},p_{2}^{m},\ldots ,p_{n}\right\}\right), \end{array}$$

where *ω* denotes the number of all possible paths *P*_*m*_ = {*p*_0_, *p*_1_^*m*^, *p*_2_^*m*^,…, *p*_*n*_} with *n* steps connecting **x** and **x**_*i*_ and totally included in the processing window *W*, *Λ*{·} is a dissimilarity value along a specific path, and *g*(·) is a smooth function of *Λ*. Finally, the DPA_last_ similarity function takes the form as follows:

(7) $$\begin{array}{c} \mu \left(x,x_{i}\right)=\mu \left(p_{0},p_{n}\right)=\overset{\omega }{\underset{m=1}{\sum }}\exp\left[-\beta \cdot \Lambda \left\{p_{0},p_{1}^{m},p_{2}^{m},\ldots ,p_{n}\right\}\right]. \end{array}$$

#### 2.3.3. Filter Output and *β* Normalization

The proposed method can use different path lengths and bit depths, and therefore, to ensure the comparability of the results, it was necessary to rescale the parameter *β*. In this case, the *β* parameter will be divided by the maximum possible cost of the single path; thus, in (5), the normalized value $\hat{\beta }$ will be used.

(8) $$\begin{array}{c} \hat{\beta }=\frac{\beta }{\max\left(\Lambda \left(P_{m}\right)\right)}. \end{array}$$

Maximum path cost can be determined using the formula as follows:

(9) $$\begin{array}{c} \text{max}\left(\Lambda \left(P_{m}\right)\right)=\left(2^{bpp}-1\right)\cdot n^{2}\cdot \sqrt{2}, \end{array}$$

where bpp denotes the number of bits per pixel and *n* the number of path steps. The next stage is a normalization of the similarity function, which can be defined as follows:

(10) $$\begin{array}{c} \psi \left(x,x_{i}\right)=\frac{\mu \left(x,x_{i}\right)}{\sum_{x_{j}\in N\left(x\right)}\mu \left(x,x_{j}\right)}. \end{array}$$

Let **x** = *p*_0_ denote the pixel under consideration, with *u*(**x**_*i*_) representing the noisy pixel **x**_*i*_; the filter output $\hat{v}\left(x\right)$ is given as follows:

(11) $$\begin{array}{c} \hat{v}\left(x\right)=\sum_{x_{i}\in N\left(x\right)}\psi \left(x,x_{i}\right)\cdot u\left(x_{i}\right). \end{array}$$

### 2.4. Extended Neighborhood and Digital Path Models

**Figure 3:**
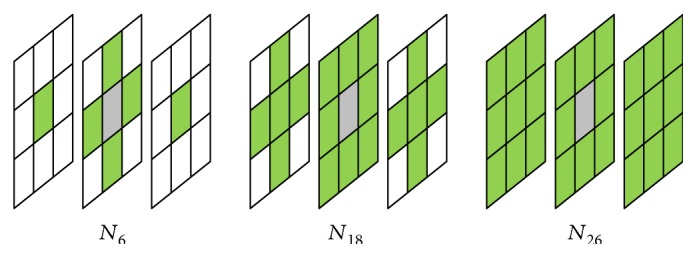
Basic spatiotemporal masks for different neighborhood systems.

**Figure 4:**
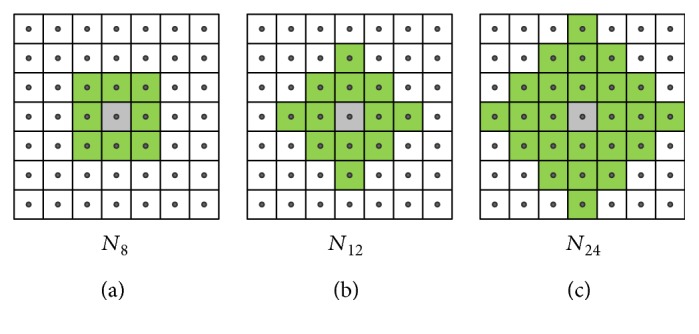
Spatial neighborhood systems utilized in our framework: (a) Standard 8 neighborhood. (b) and (c) show the extended von Neumann neighborhood with the radii 2 and 3.

**Figure 5:**
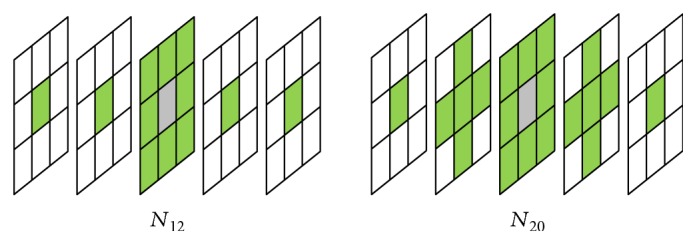
Modified spatiotemporal masks with extended neighborhood.

The selected neighborhood system significantly affects the performance of the new filters. For the static images, there are two basic types of neighborhood: *𝒩*_4_ and *𝒩*_8_, while in the case of a three-dimensional image, three spatiotemporal neighborhood systems can be defined: *𝒩*_6_, *𝒩*_18_, and *𝒩*_26_ (Figure 3). The new spatial filters with *𝒩*_8_ neighborhood and the spatiotemporal ones with *𝒩*_18_ and *𝒩*_26_ are very efficient, but they fail for heavily degraded images. Therefore, in the proposed denoising design, we introduced the extended von Neumann neighborhood [22] originally defined for cellular automata. Various neighborhood systems for static 2D images are drawn in Figure 4, while 3D neighborhoods are shown in Figure 5.

Additionally, the efficiency of the proposed denoising framework is strongly connected with the type of digital paths. Different models of paths allow us to suppress a certain type of noise [20].

**Figure 6:**
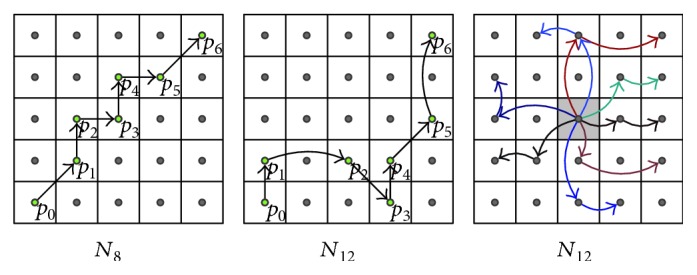
Escaping path model illustration with different neighborhood systems.

**Figure 7:**
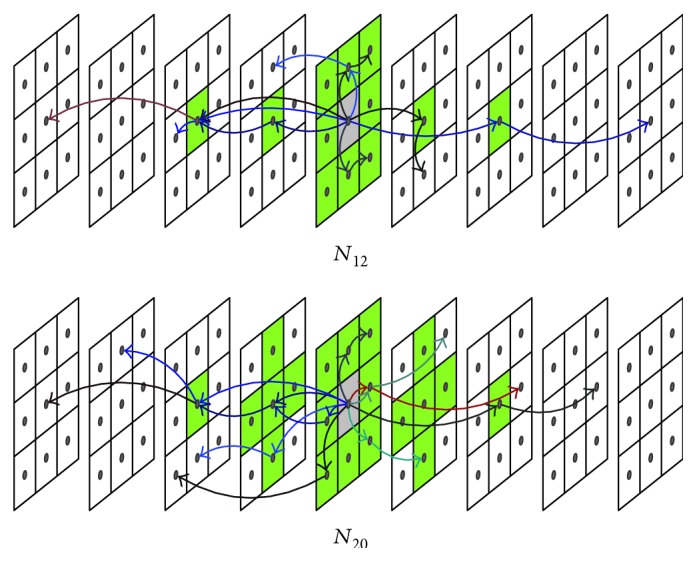
Examples of three-dimensional escaping path of length *n* = 2 limited by spatiotemporal window (spatial radius *A* = 1 and temporal radius *t* = 4).

Previous research has demonstrated that the best results are obtained in the presence of impulsive Gaussian as well as of mixed-type noise for the *self-avoiding path* (SAP) model, but our experiments suggest that in the case of ultrasound images, the greatest results are achieved for the so called *escaping path model* (EPM); thus, the new filter will be denoted as the *escaping path filter* (EPF). In the proposed denoising scheme, the topological distance from the initial point in the following steps must be increased. Exemplary spatial escaping paths created with various neighborhood systems are illustrated in Figure 6, while Figure 7 depicts the spatiotemporal case. Later in this paper, the proposed filters will be marked as EPF2D for the case of two-dimensional filtering (2D) and EPF3D for the spatiotemporal case (3D).

In situations when the images are highly contaminated, we can increase the efficiency of the filter in one of two ways: (1) extend the length of the used paths or (2) apply it in an iterative manner. The second option is much faster and more accurate and allows us to control the filter strength adjusting the *β* parameter in subsequent iterations. In this way, *β* can be updated as follows:

(12) $$\begin{array}{c} \beta \left(\kappa \right)=\beta \left(\kappa -1\right)\cdot \alpha ,\;\kappa =1,\ldots ,m. \end{array}$$

## 3. Simulation Results

### 3.1. Static Images

**Figure 8:**
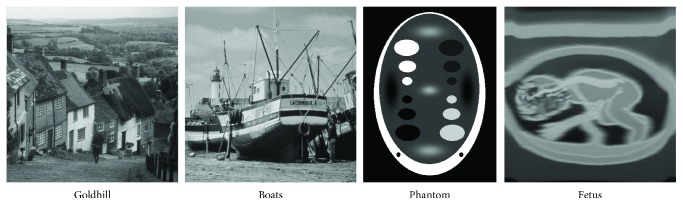
Images used for the analysis.

The commonly used benchmark images *goldhill*, *boats*, and artificially generated *phantom* were chosen to compare efficiency of different filters. Besides, we used a synthetic test image that was a phantom for a 3-month-old fetus denoted later as *fetus*. This test image was obtained using the *Field II* applications, which simulate the ultrasound field that is based on linear acoustics using the Tupholme-Stepanishen method for calculating the spatial impulse response [35–37]. The proposed ultrasound data seems to be more reliable, due to the fact that it contains artifacts typical for an ultrasound acquisition process and thus it can be used for image quality evaluation. A reference noise-free image was achieved by averaging of 500 simulated images and was depicted in Figure 8. The described filtering design has been compared with the following state-of-the-art methods capable of suppressing a speckle noise:

- Wiener filter [2]
- Speckle reducing anisotropic diffusion (SRAD) [6]
- Nonlocal means (NLM) [7, 8]
- Optimized Bayesian nonlocal means (OBNLM) [11]
- Probabilistic nonlocal means (PNLM) [9]
- Probabilistic patch-based weights (PPBW) [10]
- Digital path approach (DPA_last_) [20]

The source codes of the algorithms used in our comparison were provided by the authors of the respective papers. In order to determine the optimal values of parameters for the reference filters, we tested a wide range of parameters according to the authors' recommendations to obtain the highest possible PSNR value.

The recommended values of the parameters *α* and *β* for the proposed technique were adjusted, so that the best PSNR value was achieved. The test images were deteriorated by the multiplicative noise described by (1) with mean *μ* = 0 and *σ*^2^ = 0.2, 0.4, and 0.6 except for the *fetus* image, which was contaminated using a more realistic artifacts resulting from the physical model of an ultrasound image acquisition process. The restoration efficiency has been assessed using the peak signal-to-noise ratio (PSNR) and more sophisticated mean structural similarity index (MSSIM) calculated with Gaussian kernel and default parameters [38].

**Figure 9:**
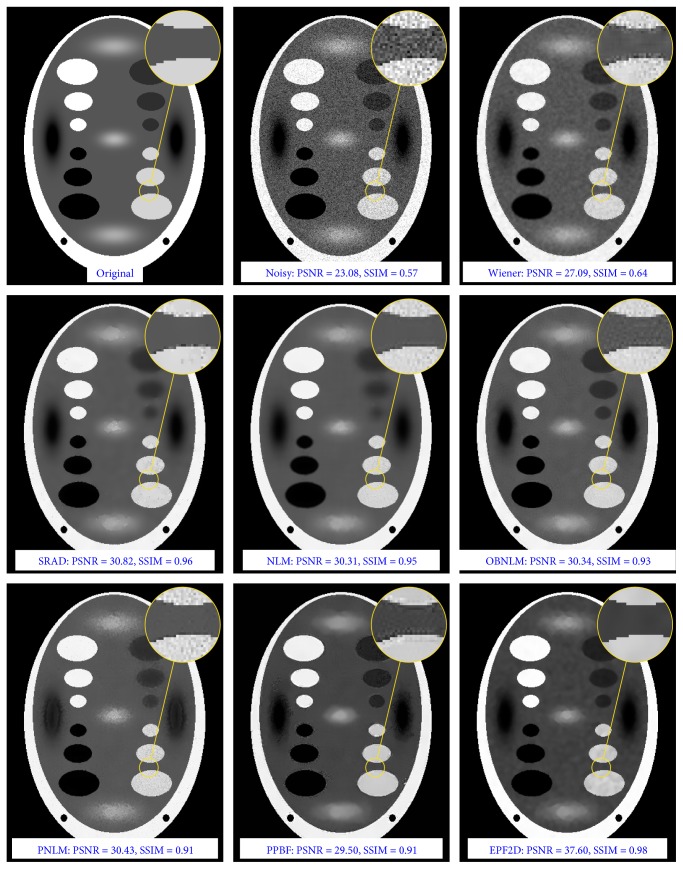
Visual comparison of the filtering efficiency of the artificial *phantom* image deteriorated by speckle noise with *σ*^2^ = 0.4.

Numerical results obtained for the static images are summarized in Table 1. The conducted simulations revealed that the proposed EPF approach outperforms other techniques for highly deteriorated real images in terms of PSNR metric, while algorithms that are based on nonlocal means provide slightly better results for lower noise contamination level. Additionally, the proposed filter gives better results for synthetic images. A visual comparison of the results achieved for the phantom image is drawn in Figure 9. From this figure, it can be seen that most filters removed multiplicative noise and produces more visually pleasing results, but the outcomes are slightly blurred.

**Figure 10:**
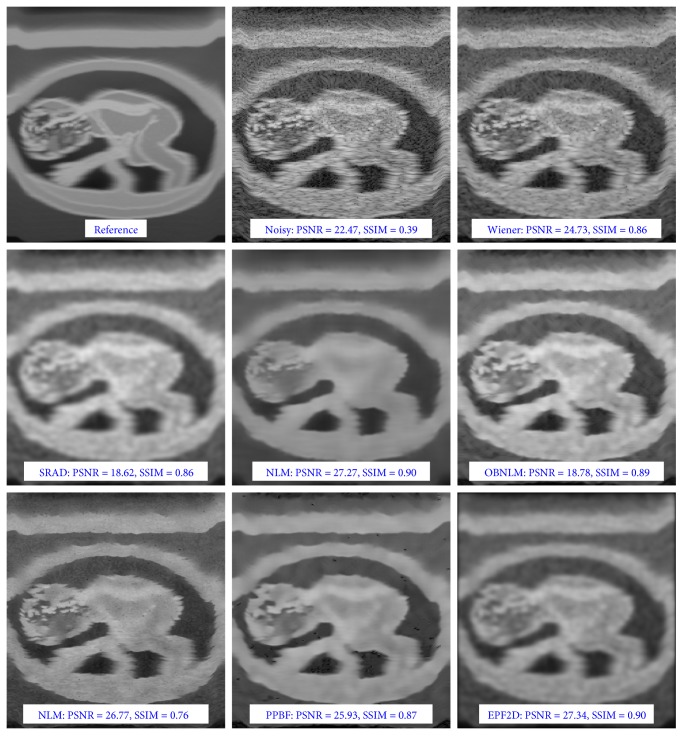
Visual comparison of the performance of the filtering algorithms for the *fetus* image obtained using the *Field II* application.

**Figure 11:**
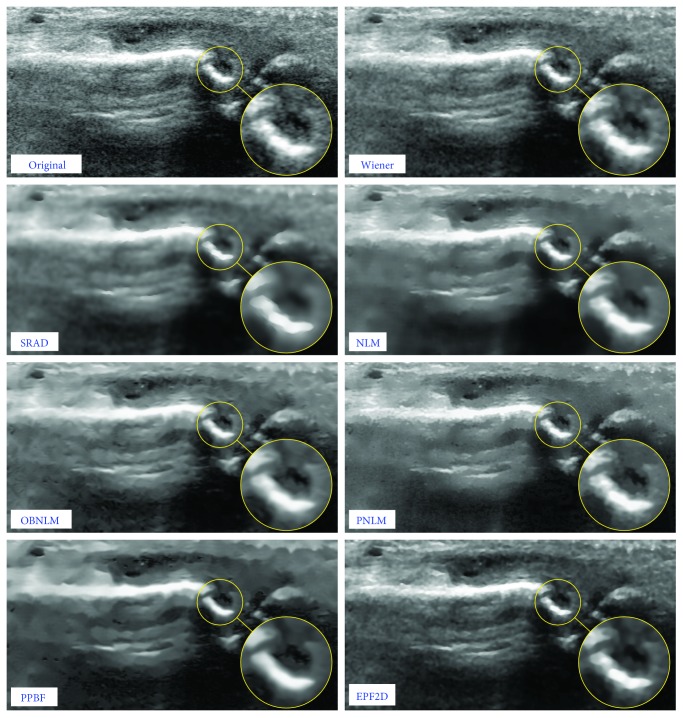
Visual comparison of the filtering efficiency evaluated on a real ultrasound image contaminated by multiplicative noise.

The most significant and valuable results seems to be obtained for simulated fetus image, because it is much similar to the realistic ultrasound images. The visual comparison of the performance of the analyzed filters for the *fetus* image is presented in Figure 10, while exemplary results for the real ultrasound images of fingers are depicted in Figure 11. The assessment of the achieved results can be also evaluated by segmentation accuracy applying image denoising as the preprocessing step, but currently, it is out of scope in this work. The proposed denoising scheme requires also a much smaller neighborhood than that used in family of nonlocal means methods; therefore, our approach is much faster and less aggressively blurs the image. It should also be emphasized that for all methods based on the concept of NLM, even a small modification of optimal values of parameters gives a significant decrease in performance, while the EPF framework gives acceptable results for a wide spectrum of parameters.

### 3.2. Video Sequences

The images obtained by ultrasound devices are already enhanced by built-in filtering algorithms, but the resulting effect may still be unsatisfactory. So we can add another stage of image enhancement. Such technique should be capable of real-time processing. The *escaping path filters* are close to satisfy this condition on standard PC. Additionally, those filters are suitable for GPU implementation because all paths can be calculated in parallel and it lacks branches which block GPU threads. Our preliminary experiments show that we can obtain over 50 FPS for standard CIF sequences. Thus, several filters capable of real-time video processing were compared with our new approach. Additionally more complex techniques, based on nonlocal means [8] and BM3D [39], were added to comparison; however, the computational complexity of those filters limits their use for offline processing. The performance of the following filters was evaluated:

- Wiener 2D—a spatially adaptive Wiener filter
- Wiener 3D (3 × 3 × 3),
- Temporal Gaussian filter (TGauss) (*n* = 5 and *σ* = 5)
- Spatiotemporal median filter (3 × 3 × 3)
- Nonlocal means denoising [8] and our spatiotemporal implementation (NLM3D)
- Block-matching and 3D filtering (BM3D) [39]
- Spatial fast digital path approach (FDPA) (*β* = 15) [20]
- Spatiotemporal fuzzy FDPA filter (STFFDPA) (*n* = 5, *σ* = 5, *γ* = 4, and *β* = 15) [18]
- Spatial escaping path filter EPM_1st_2D (*𝒩*_12_),
- Escaping path spatiotemporal filters EPM_1st_3D and EPM_last_3D (*𝒩*_20_)

The video denoising algorithms were tested using publicly available video sequences: *foreman*, *salesman*, and *tennis*, contaminated by the multiplicative noise described in (1) with mean *μ* = 0 and *σ*^2^ = 0.2, 0.4, and 0.6. To obtain a more realistic comparison, we have prepared a test sequence based on the *fetus* image. Base fetus sequence consists of 150 frames subjected to different transformations that simulate the possible displacements during the ultrasound acquisition process. Then, all the frames have been subjected to a simulation using the *Field II* application. Virtually noise-free reference video was obtained by averaging of 200 simulation results (reference videos could be downloaded from http://dip.aei.polsl.pl/usg/fetus_video.7z).  Table 2 summarizes results obtained for the set of test video sequences. Based on synthetic tests only, it is difficult to choose the best filter. In most cases, especially for small levels of noise, the best PSNR results are obtained for the BM3D filter; however, our spatiotemporal solution gives only slightly worse results at much higher processing speed. For heavily corrupted sequences, we can clearly see the advantage of the proposed filtrating technique. It should be noted that for the more realistic ultrasound noise model, obtained using the *Field II* application, the advantages of our solution is clear (the best results were obtained for the NLM3D filter, but the computational complexity disqualifies it entirely, even for offline processing). In the case of the *tennis* sequence with minimal disruption, most filtering techniques deteriorate quality ratios. This is due to the specific background that resembles an impulsive noise pattern.  Figure 2() presents exemplary filtering results and SSIM maps for the mentioned sequence. It is thus clear that the worst results were obtained for filtering based on the median, which effectively removes elements of background texture. In addition, median 3D filtering introduces some temporal artifacts, such as blurred or erased moving objects. It should be noted that the ranking obtained with the SSIM index is slightly different in favor of the escaping path filters.

## 4. Conclusions

In this paper, a new class of fast spatial and spatiotemporal filters was presented. The proposed filtering techniques were designed for multiplicative noise suppression, specifically for ultrasound image and video filtering. The novel approach is based on the special type of digital paths, the so called *escaping paths*, created on an image lattice—spatial in a 2D case or spatiotemporal for video processing. Additionally, the new extended neighborhood model was introduced, based on von Neumann concept derived from cellular automata theory. The presented methods give comparable or better results to the other methods, both for static image and video sequences. Another beneficial feature of the proposed denoising scheme is its lower computational complexity than that of other state-of-the-art techniques, which allows us to apply it in real-time image processing tasks. The proposed filter is also more stable for a wide range of input parameters and gives satisfactory results in terms of different quality metrics and visual inspection.

## Figures and Tables

**Figure 12:**
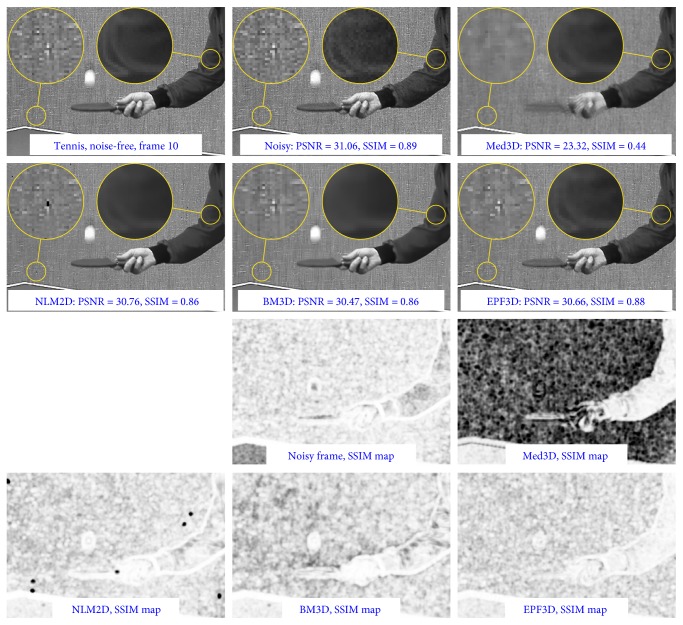
Denoising results of frame 10 from the *tennis* video sequence corrupted with multiplicative noise (*σ*^2^ = 0.2) and the corresponding SSIM quality maps.

**Table 1:** Comparison of the efficiency of the proposed filter and competitive methods using the PSNR and MSSIM quality measure on the static images.

File noise (*σ*^2^)	Goldhill			Boats			Phantom			Fetus
	0.2	0.4	0.6	0.2	0.4	0.6	0.2	0.4	0.6	
PSNR results (dB)										
Noisy	31.52	19.69	13.25	30.65	18.75	12.62	35.05	23.03	16.48	22.46
Wiener	30.76	27.09	22.24	31.01	26.69	21.90	37.36	28.57	21.19	24.73
SRAD	30.73	27.26	23.69	32.06	26.96	23.98	42.52	30.82	24.14	18.62
NLM	34.06	27.35	16.49	35.40	27.68	15.54	43.07	30.31	20.15	27.27
OBNLM	30.87	28.24	24.43	32.61	28.06	24.13	38.64	30.34	23.76	18.78
PNLM	34.20	27.18	22.75	34.52	27.34	22.13	42.21	30.43	23.00	26.77
PPBF	32.80	27.11	18.56	32.64	26.91	20.00	37.01	29.50	21.15	25.93
DPA_last_	32.83	27.77	24.96	32.61	27.10	24.20	44.98	34.65	26.63	26.69
EPF2D	33.63	27.98	25.24	33.48	27.49	24.21	48.57	37.60	27.32	27.34
MSSIM results										
Noisy	0.83	0.32	0.11	0.75	0.30	0.13	0.86	0.57	0.47	0.39
Wiener	0.77	0.64	0.43	0.84	0.67	0.43	0.98	0.91	0.67	0.66
SRAD	0.88	0.67	0.54	0.92	0.72	0.65	0.99	0.96	0.89	0.86
NLM	0.88	0.65	0.20	0.93	0.69	0.25	0.99	0.95	0.56	0.90
OBNLM	0.90	0.69	0.56	0.93	0.69	0.63	1.00	0.93	0.79	0.84
PNLM	0.90	0.64	0.42	0.92	0.67	0.39	0.98	0.91	0.80	0.76
PPBF	0.86	0.69	0.35	0.88	0.74	0.53	0.95	0.89	0.68	0.87
DPA_last_	0.87	0.69	0.59	0.89	0.73	0.59	0.99	0.95	0.88	0.85
EPF2D	0.90	0.70	0.59	0.90	0.74	0.61	1.00	0.95	0.93	0.90

**Table 2:** Comparison of the filtering algorithms applied for standard test videos corrupted with different noise scenarios.

Video sequence noise *σ*^2^	Foreman			Salesman			Tennis			Fetus
	0.2	0.4	0.6	0.2	0.4	0.6	0.2	0.4	0.6	
PSNR results (dB)										
Noisy	30.23	18.70	12.52	33.83	21.83	15.42	31.13	19.20	12.65	23.17
Wiener 2D	33.87	24.00	19.70	33.00	25.94	19.70	29.93	23.92	16.61	24.12
Wiener 3D	33.90	24.24	20.00	32.75	26.18	20.00	29.56	24.12	17.57	26.86
TGauss	30.60	23.30	20.61	33.56	26.50	20.61	27.56	22.50	17.32	26.30
Median 3D	30.00	26.65	24.51	29.15	27.48	24.51	21.70	21.21	19.64	26.36
NLM2D	35.05	27.92	22.28	34.37	26.88	22.28	25.96	26.39	17.75	29.10
NLM3D	35.67	28.02	22.34	32.79	27.06	22.34	30.75	27.64	17.81	30.28
BM3D	34.44	30.93	23.12	35.25	32.30	24.92	31.97	28.63	22.64	28.40
FDPA	32.74	25.32	20.23	31.28	27.24	20.24	27.40	24.09	16.73	24.80
STFFDPA	33.47	23.09	18.25	33.37	26.16	18.24	28.91	23.06	15.06	24.51
EPF2D	33.86	25.69	20.99	32.16	27.64	24.07	28.50	24.36	19.89	25.76
EPF_1st_3D	34.39	28.05	25.02	34.40	29.28	25.02	30.85	27.20	20.99	28.92
EPF_last_3D	33.27	28.10	25.05	34.28	29.07	25.06	30.11	25.85	21.20	29.34
MSSIM results										
Noisy	0.72	0.25	0.09	0.90	0.48	0.22	0.82	0.35	0.16	0.40
Wiener 2D	0.89	0.47	0.41	0.90	0.69	0.41	0.78	0.55	0.24	0.49
Wiener 3D	0.89	0.47	0.43	0.90	0.70	0.43	0.78	0.56	0.26	0.64
TGauss	0.81	0.40	0.41	0.92	0.67	0.41	0.82	0.49	0.25	0.61
Median 3D	0.84	0.63	0.59	0.86	0.76	0.59	0.59	0.47	0.35	0.75
NLM2D	0.92	0.71	0.50	0.91	0.73	0.50	0.85	0.68	0.30	0.90
NLM3D	0.92	0.71	0.50	0.88	0.73	0.50	0.86	0.69	0.31	0.91
BM3D	0.90	0.83	0.52	0.93	0.89	0.60	0.81	0.69	0.48	0.86
FDPA	0.87	0.55	0.42	0.88	0.74	0.42	0.72	0.54	0.24	0.54
STFFDPA	0.89	0.44	0.33	0.92	0.69	0.33	0.79	0.49	0.21	0.52
EPF2D	0.90	0.58	0.34	0.90	0.77	0.57	0.78	0.59	0.35	0.64
EPF_1st_3D	0.91	0.72	0.64	0.93	0.83	0.63	0.86	0.69	0.40	0.88
EPF_last_3D	0.89	0.71	0.60	0.93	0.82	0.60	0.84	0.65	0.41	0.89
